# Data mining combined with experiments to validate CEP55 as a prognostic biomarker in colorectal cancer

**DOI:** 10.1002/iid3.375

**Published:** 2020-11-15

**Authors:** Kang Lin, Xiaojian Zhu, Chen Luo, Fanqin Bu, Jinfeng Zhu, Zhengming Zhu

**Affiliations:** ^1^ Department of Gastrointestinal Surgery The Second Affiliated Hospital of Nanchang University Nanchang Jiangxi China; ^2^ The Seventh Affiliated Hospital of Sun Yat‐sen University Shenzhen China

**Keywords:** bioinformatics analysis, biomarkers, CEP55, colorectal cancer, p53/p21 axis, prognosis

## Abstract

**Introduction:**

Colorectal cancer (CRC) is a common tumor with high morbidity and mortality. Current specific diagnosis regarding CRC remains complicated and costly, and specific diagnostic biomarkers are lacking.

**Methods:**

To find potential diagnostic and prognostic biomarkers for CRC, we screened and analyzed many CRC sequencing data by The Cancer Genome Atlas Program and Gene Expression Omnibus, and validated that CEP55 may be a potential diagnostic biomarker for CRC by molecular cytological experiments and immunohistochemistry, among others.

**Results:**

We found that CEP55 is upregulated in CRC tissues and tumor cells and can promote CRC proliferation and metastasis by activating the p53/p21 axis and that CEP55 mutations in tumor patients result in worse overall survival and disease‐free survival time. Besides, we also found that genes, such as CDK1, CCNB1, NEK2, KIF14, CDCA5, and RFC3 were upregulated in tumors, and their mutations would affect the prognosis of CRC patients, but these results await for more experimental evidence.

**Conclusion:**

Our study validates CEP55 as a potential diagnostic and prognostic biomarker for CRC, and we also provide multiple genes and potential molecular mechanisms that may serve as diagnostic and prognostic markers for CRC.

## Graphical abstract

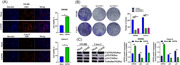

## 1 INTRODUCTION

According to the latest statistics from the American Cancer Society, colorectal cancer (CRC) patients have become the second most common cause of cancer death in the United States.^1^ Moreover, more than half of all deaths from CRC are due to modifiable risk factors.^2^ Somatic and epigenetic abnormalities caused by these risk factors dominate.^3^ Hence, how to screen the risk of CRC patients has become an urgent matter to be resolved. The current screening and diagnosis methods for CRC mainly include colonoscopy, computed tomography colonoscopy, tool testing, and biomarkers.^4^ After considerable research progress, there have been studies showing that abnormal expression and mutation of genes are related to the carcinogenicity and progress of CRC. These mutations include mutation inactivation of tumor suppressor genes, the most common is germline APC mutations, and p53 pathway inactivation caused by TP53 mutations.^5^ Most of the research focuses on a few markers, such as TP53, KRAS, BRAF, GNAS mutations, or some genes related to this pathways.^6^ The current study has not clarified the specific diagnosis and prognostic biomarkers of CRC, which may be due to inadequate sample quality or quantity, the high cost of screening mutations, or the lack of standardized recruitment of patients and timely follow‐up.^7^ All these factors lead to the difficulty and accuracy requirements of this study.

Biomarkers are significant clinically. In this study, we used large‐scale transcriptome data in The Cancer Genome Atlas Program (TCGA) and Gene Expression Omnibus (GEO), through standard computer algorithms, to screen some genes that had previously unknown roles in CRC, by analyzing their expression levels and correlation with some standard clinical‐pathological features. Combined with molecular biology and cytology experiments to verify a gene that has never been reported in CRC before, and verified a mechanism for regulating CRC.

## 2 MATERIALS AND METHODS

### 2.1 Public database and experimental materials

GEO (https://www.ncbi.nlm.nih.gov/geo/)^8^ is a public genomic database that plays an important role in cancer research and contains a wealth of data. Three gene expression datasets (GSE110225,^9^ GSE22598,^10^ GSE37364^11^) were downloaded from GEO (Affymetrix GPL570 platform, Affymetrix Human Genome U133 Plus 2.0 Array). According to the annotation information in the platform, the probes were converted into the corresponding gene symbol. The GSE110225 dataset contained 34 samples (17 CRC samples and 17 noncancerous samples). The GSE22598 dataset contained 38 samples (we selected 17 pairs of CRC and noncancerous tissues out of 38 samples). The GSE37364 dataset contained 94 samples (we selected 27 pairs of CRC and noncancerous tissues to carry out the next research). We also downloaded 437 CRC samples (398 tumor samples and 39 para‐cancer samples) sequencing data from the TCGA database to screen for differential expression genes. CRC cell lines HT‐29, HCT116, SW480, LOVO, and Caco‐2 are all from American Type Culture Collection. All cells were cultured in Dulbecco's modified Eagle's medium supplemented with 10% fetal bovine serum (Gibco), 1% penicillin, and 1% streptomycin (Life Technologies).

### 2.2 Data processing and screening hub gene

Data preprocessing includes background adjustment, normalization, and summarization. We downloaded the expression matrix files and the corresponding platform files for these three datasets separately. When probe information was converted into gene symbol, they were preprocessed by the affy package of R software.^12^ Then the limma package^13^ was used to identify the upregulated and downregulated differential expression genes (DEGs) between CRC and healthy controls. Adjusted *p* values (adj. *p*) and Benjamini and Hochberg false discovery rates were used to maintain a balance between limitations of false‐positives and the discovery of statistically significant genes. The cutoff criteria were set at |log2(Fold change)| > 1 and adj. *p* < .01 after excluding genes without a gene symbol and having multiple probe sets. The results of the final DEGs for each data set were screened. Then take the intersection of these DEGs through the Venn diagram. We standardized the data downloaded from TCGA with the limma package and analyzed the DEGs in tumor tissues and adjacent tissues. Combined with the screening results of GEO, and draw a volcano map of DEGs. We analyzed the protein interactions of the 284 differential genes after screening in STRING (https://string-db.org/), and then counted the genes with more than ten nodes in these networks as the Hub genes. The functional enrichment analysis of these genes was subsequently conducted in Metascape to analyze their potential molecular mechanisms involved in the regulation of CRC.

### 2.3 Construct a protein–protein interaction network and screen key modules

STRING (version 11.0)^14^ database is an online database that can be used to retrieves known protein‐protein interaction (PPI) information or predicts PPI information. PPI network of DEGs was built using the STRING database in the present study. We mapped the DEGs to STRING, only experimentally validated interactions with a combined score greater than 0.4 are considered significant. Then, using the Cytoscape (version 3.7.0)^15^ software to construct PPI networks. Molecular Complex Detection (MCODE) (v1.5.1)^16^ is a Cytoscape app, which can be used to screen the significant module in the PPI networks. The parameters are set as follows: MCODE scores >5, degree cut‐off = 2, node score cut‐off = 0.2, Max depth = 100 and *k* score = 2. *p* < .05 was considered statistically significant.

## 3 HUB GENE SELECTION AND ANALYSIS

After analyzing the protein interactions of these DEGs, we analyzed the node information of each gene in the network, sorted them according to the size of the connection flux, and finally selected the gene with a node connection score of ≥10 as Hub gene. The cBioPortal (http://www.cbioportal.org),^17^, ^18^ an online platform, was used to analyze the co‐expression genes and genes' network. Biological Networks Gene Oncology tool (BiNGO) (version 3.0.3),^19^ another useful app of Cytoscape, was used to perform and visualize biological process analysis of hub genes. We then used the Xena Functional Genomics Explorer (https://xenabrowser.net/heatmap)^20^ to construct the hierarchical clustering of hub genes. The overall survival (OS) and disease‑free survival (DFS) analyses of these hub genes have been performed by using the Kaplan–Meier curve in the cBioPortal.

### 3.1 The expression of CEP55 in multiple databases

CEP55 expression was compared between tumors and healthy in the Oncomine database. By downloading and analyzing transcriptome data from 437 CRC samples in TCGA, we extracted the expression levels of CEP55 and plotted violin plots, and a *t* test was used to analyze the statistical index *p* value. We also analyzed the expression of CEP55 in tumor tissues and healthy tissues of human organs in GEPIA2 (http://gepia2.cancer-pku.cn/#index) database^21^ and analyzed the expression of CEP55 in different human tissues. Subsequently, we analyzed the correlation of CEP55 expression with tumor type, tumor stage, age, and lymph node metastasis using the UALCAN (http://ualcan.path.uab.edu/) database.^22^

### 3.2 Experimentally verified that CEP55 regulates CRC progression

We verified the expression of CEP55 in CRC through some experimental techniques, such as reverse transcription‐polymerase chain reaction (RT‐PCR), immunohistochemistry, and Western blot analysis. The function of CEP55 was then analyzed, and the relevant molecular pathways involved in the CEP55 regulation of CRC were searched. The detailed experimental procedures were approximately the same as in the study by Ming Zhong et al.^23^, ^24^

## 4 RESULTS

### 4.1 Screen hub genes and perform functional analysis

**Figure 1:**
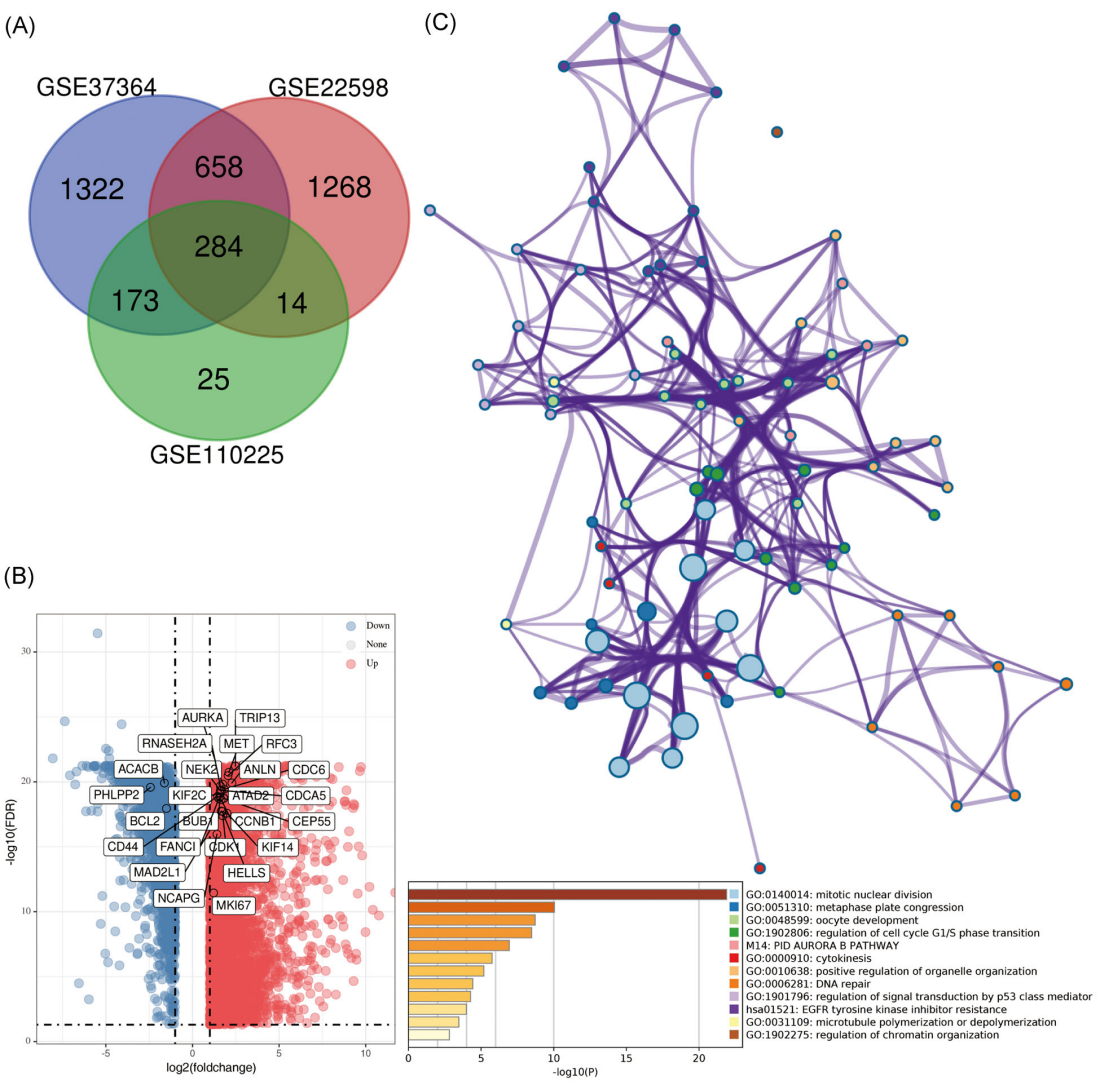
Screening differential expression genes (DEGs) and enrichment analysis of Hub genes. (A) Venn diagram. DEGs were selected with a |log2 (fold change)| > 1 and adjust*p* < .01 among the messenger RNA (mRNA) expression profiling sets GSE110225, GSE22598, and GSE37364. The three datasets showed an overlap of 284 genes. (B) Volcano graph. Differential analysis was performed on 437 colorectal cancer (CRC) samples (398 tumor tissues and 39 paired tissues adjacent to cancer) in The Cancer Genome Atlas Program (TCGA), and 6500 differentially expressed genes were screened (4477 upregulated and 2023 downregulated). (C) The top 12 GO and pathway cluster networks obtained by enrichment analysis of the 28 selected target genes

**Figure 2:**
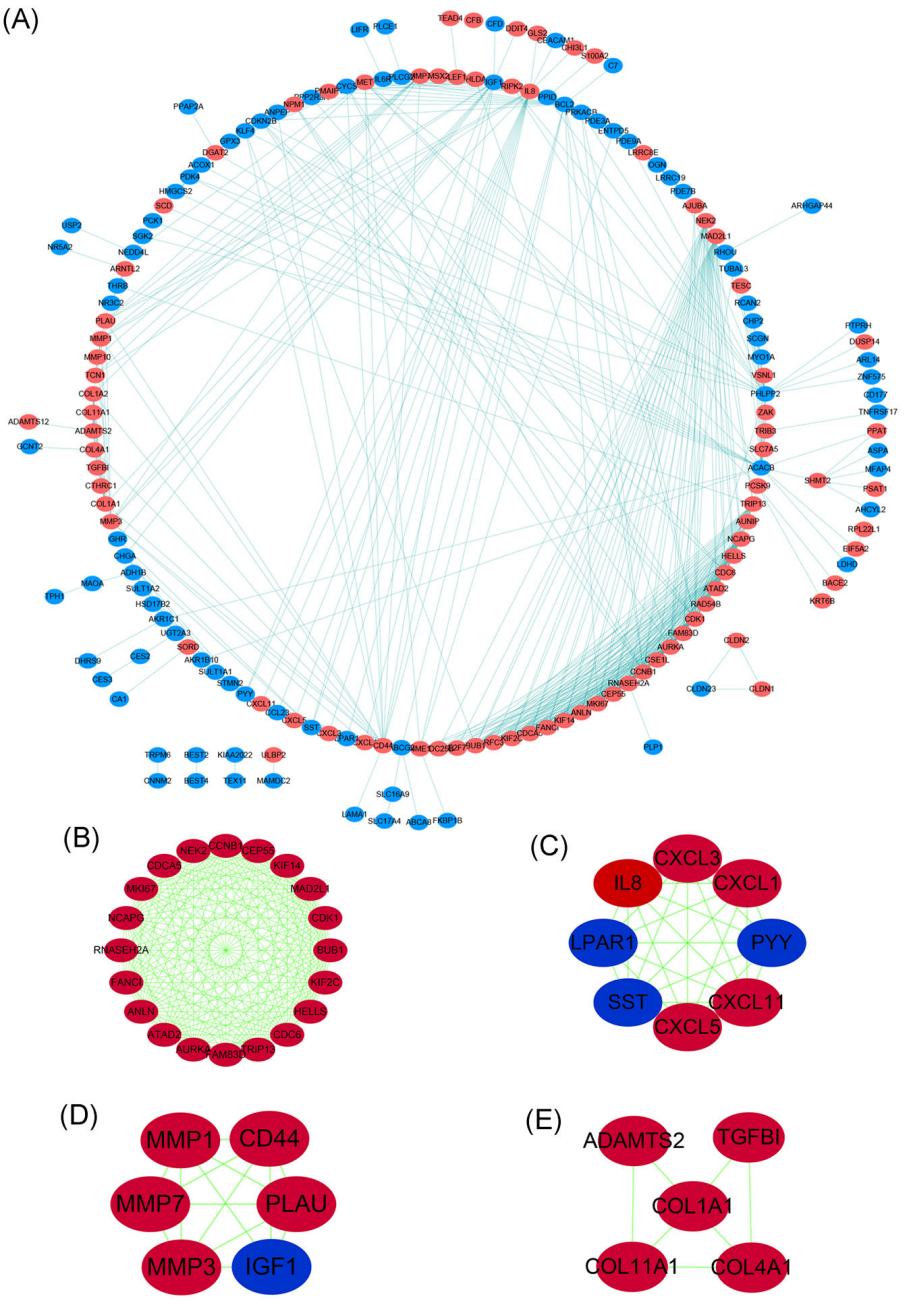
The protein–protein interaction (PPI) network of DEGs and the most important module constructed by Cytoscape. (A) PPI network. Upregulated genes are marked with orange–red; downregulated genes are marked with dark turquoise. Genes associated with each other are marked with dark turquoise lines. (B–E) Four highly connected clusters were obtained from the PPI network. Upregulated genes are marked with dark red; downregulated genes are marked with dark blue. DEG, differential expression gene

By performing a differential analysis of three data sets in GEO, we obtained a series of DEGs. After using the Venn diagram to perform the intersection operation, 284 common DEGs (160 downregulated and 124 upregulated) were finally determined (Figure 1A). We analyzed the interaction of these genes in STRING and scored each gene according to the number of associations with other genes from high to low. Eventually, 28 genes with scores ≥10 were selected as Hub genes in this study. Some of their primary functions and related information were shown in Table 1. By analyzing the CRC transcriptome data in TCGA through a computer algorithm, we obtained a series of DEGs and plotted a volcano map (Figure 1B). We conducted functional enrichment analysis to preliminary analyze the functions of these 28 Hub genes in tumors (Figure 1C). The analysis results show that the biological functions of these genes mainly involved in tumors include mitotic nuclear division, metaphase plate congression, regulation of cell cycle G1/S phase transition, and other processes related to cell replication and cell cycle. Second, we also found that the Kyoto Encyclopedia of Genes and Genomes^25^ pathway they participated in was mainly related to epidermal growth factor receptor (EGFR) tyrosine kinase inhibitor resistance, phosphoinositol‐3‐kinase (PI3K)‐Akt signaling pathway, and p53 signaling pathway. The PPI network of DEGs was constructed with STRING and Cytoscape (Figure 2A). Meanwhile, the most significant module was selected by using the MCODE plug‐in of Cytoscape (Figure 2B–E). The results show that the Hub genes we screened are mainly the genes upregulated in CRC.

**Table 1:** Functional roles of 28 hub genes

No.	Gene symbol	Degree	Full name	Function
1	CDK1	31	Cyclin dependent kinase 1	CDK1 can regulate the cell cycle progression, apoptosis, and carcinogenesis of tumor cells.
2	CCNB1	29	Cyclin B1	CCNB1 is linked to invasion, metastasis, and poor prognosis of several cancers.
3	AURKA	27	Aurora kinase A	AURKA plays a crucial role in tumor progression.
4	PHLPP2	25	PH domain and leucine rich repeat protein phosphatase 2	The expression of PHLPP2 is related to colorectal cancer and bladder urothelial carcinoma. Among its related pathways, RET signaling and PI3K/AKT activation are included.
5	MAD2L1	23	Mitotic arrest deficient 2 like 1	MAD2L1 is involved in multiple cellular functions, such as translation DNA synthesis, signal transduction, transcription, and mitotic events.
6	BUB1	23	BUB1 mitotic checkpoint serine/threonine kinase	BUB1 is involved in mitosis by encoding serine/threonine‐protein kinase, which also plays an important role in DNA damage response. Meanwhile, this gene variant is associated with a variety of cancers.
7	NCAPG	23	Non‐SMC condensin I complex subunit G	NCAPG is overexpressed in melanomas and gliomas. Meanwhile, it was downregulated in out‐of‐niche primary tumor cells from multiple myelomas and acute myeloid leukemias.
8	CDC6	23	Cell division cycle 6	Overexpression of CDC6 had been shown to contribute to oncogenesis. The upregulations of it were linked to cancer progression in various types of cancer.
9	TRIP13	23	Thyroid hormone receptor interactor 13	High expression of TRIP13 is associated with serval types of human cancers. Meanwhile, it regulates tumor cell proliferation, migration and invasion.
10	IL8	23	C‐X‐C motif chemokine ligand 8	CXCL8 mediate the initiation and development of various cancers, such as breast cancer, prostate cancer, lung cancer, colorectal carcinoma, and melanoma.
11	ACACB	23	Acetyl‐CoA carboxylase beta	ACACB is significantly overexpress in adipose tissue, and controls fatty acid oxidation. Meanwhile, it is associated with biotin deficiency and diabetes mellitus, noninsulin‐dependent.
12	KIF2C	21	Kinesin family member 2C	KIF2C is essential in the regulation of cellular senescence and contributes to tissue/organism aging and protection of cellular transformation.
13	CEP55	20	Centrosomal protein 55	High expression of CEP55 can promote the proliferation of lung, breast and thyroid cancers.
14	FANCI	20	FA complementation group I	FANCI, one gene of the Fanconi anemia complementation group, alternative splicing results in two transcript variants encoding different isoforms.
15	NEK2	20	NIMA related kinase 2	NEK2 is highly expressed in various tumor types and cancer cell lines which is associated with rapid relapse and poor outcome in multiple cancer types.
16	IGF1	19	Insulin like growth factor 1	IGF1 is involved in mediating growth and development of cells. Meanwhile, it is overexpressed in endometrium, liver and prostate.
17	ATAD2	18	ATPase family AAA domain containing 2	ATAD2 is an emerging oncogene that has strongly been linked to the etiology of multiple advanced human cancers. Furthermore, it may potentially be utilized as a promising target for future development of RNAi‐based therapy to treat cancers.
18	ANLN	18	Anillin actin binding protein	ANLN was overexpressed in CRC, and the expression of ANLN was linked to tumor invasion and enlarged tumor size.
19	FAM83D	18	Family with sequence similarity 83 member D	FAM83D expression is elevated in hepatocellular carcinoma, ovarian cancer and metastatic lung adenocarcinomas. Furthermore, it can be a potential oncogene for many human cancer types.
20	BCL2	18	BCL2 apoptosis regulator	BCL2 was significantly overexpressed in thyroid, and blocked the apoptotic death of lymphocytes that was thought to be the cause of follicular lymphoma.
21	HELLS	17	Helicase, lymphoid specific	Overexpressed HELLS is significantly associated with liver cancer and related to poor prognosis in patients.
22	MKI67	17	Marker of proliferation Ki‐67	MKI67 encodes a nuclear protein that is related to and may be necessary for cellular proliferation. Breast cancer will be marked with high expression of MKI67.
23	CD44	17	CD44 molecule (Indian blood group)	CD44 is involved in lymphocyte activation and circulation. Besides, it is associated with tumor cell metastasis.
24	KIF14	16	Kinesin family member 14	KIF14 was identified as a likely oncogene in breast, ovarian and lung cancers, as well as retinoblastomas and gliomas.
25	CDCA5	15	Cell division cycle associated 5	Malignant progression was promoted by the upregulation of CDCA5 in urothelial carcinoma, lung cancer and oral squamous cell carcinoma, and predicted poor prognosis.
26	RFC3	14	Replication factor C subunit 3	Upregulation of RFC3 promotes the metastasis, progression, and invasion of triple‐negative breast cancer.
27	RNASEH2A	13	Ribonuclease H2 subunit A	High expression of RNaseH2A is involved in the progression of human gliomagenesis and kidney cancers.
28	MET	12	MET proto‐oncogene, receptor tyrosine kinase	MET mutations and amplification are associated with a variety of human cancers, such as papillary renal cell carcinoma and Hepatocellular carcinoma.

Abbreviations: PI3K, phosphoinositol‐3‐kinase; RET, rearranged during transfection.

### 4.2 Construct a coexpression network and mutation survival analysis

**Figure 3:**
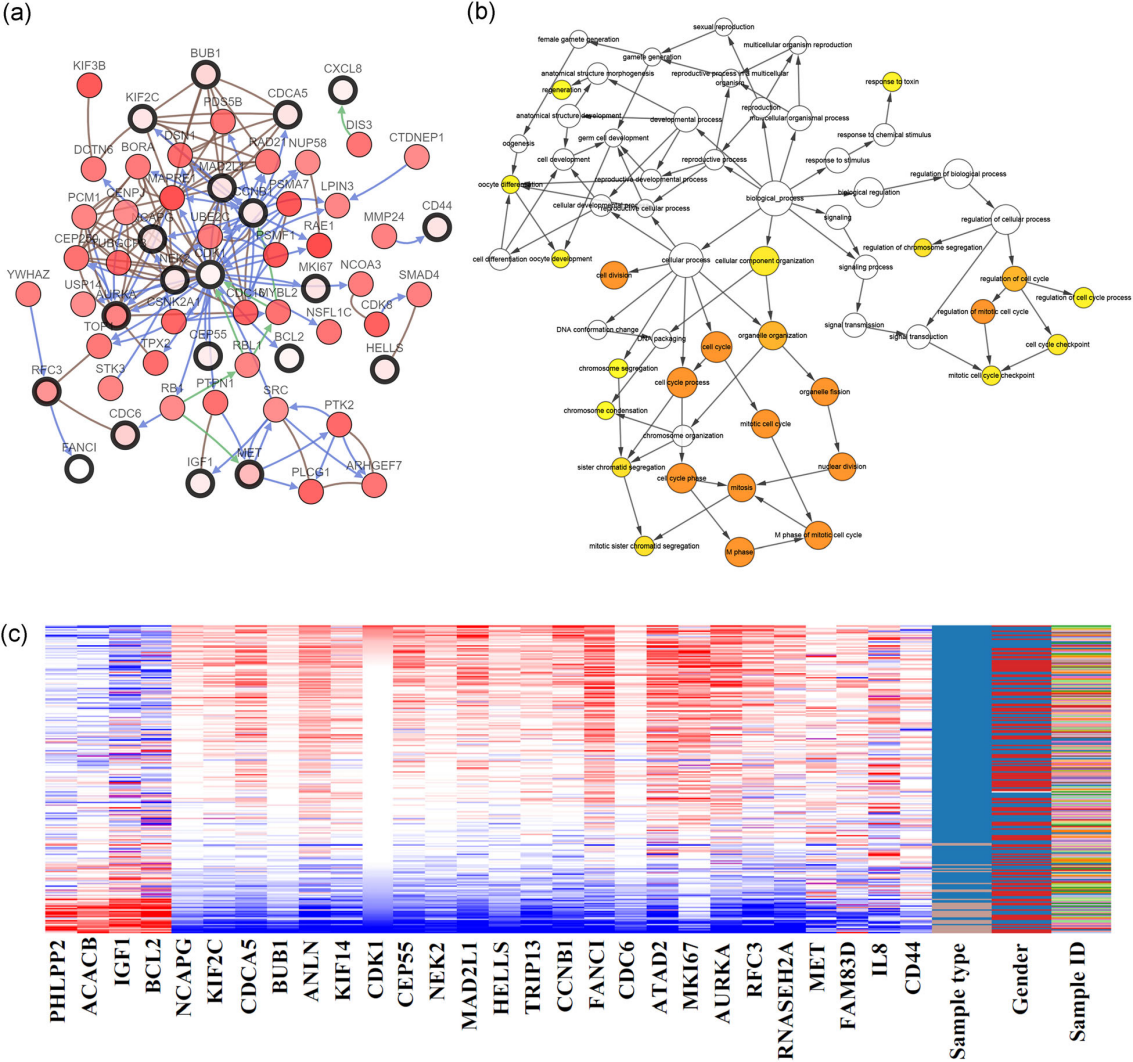
Interaction network and biological process analysis of the hub genes. (A) Hub genes and their coexpression genes were analyzed with cBioPortal. Hub genes were represented with a bold black outline. The coexpression genes were represented with a thin black outline. (B) The biological process analysis of hub genes was constructed with the BiNGO plug‐in of Cytoscape. The color depth of nodes represents the corrected*p*value of ontologies. The size of the nodes represents the number of genes that are involved in the ontologies.*p* < .001 was considered to have statistical significance. (C) Use Xena Functional Genomics Explorer to construct the hierarchical clustering of hub genes. The samples under the pink bar are noncancerous, and under the blue bar are CRC samples. The upregulation of genes marked with red, downregulation of genes marked with blue. CRC, colorectal cancer

**Figure 4:**
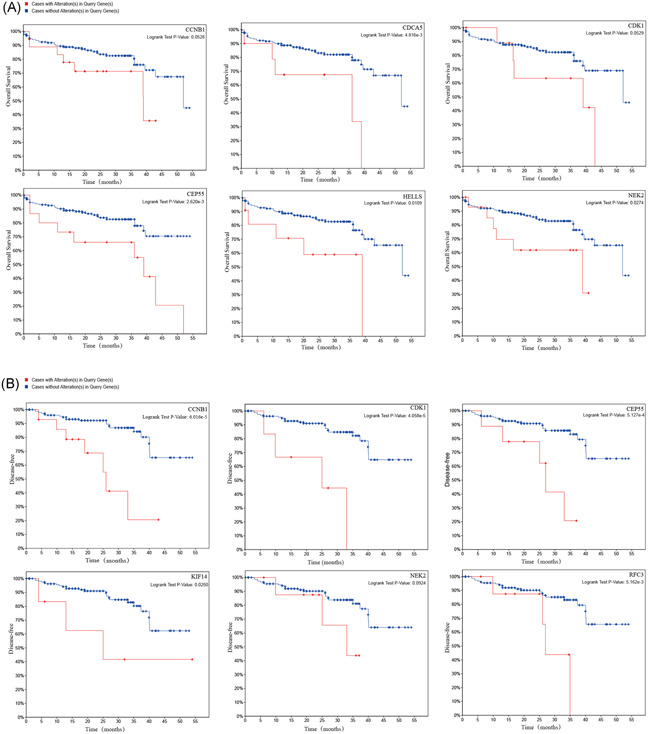
Survival analysis of hub gene. (A) Overall survival analyses of hub genes and (B) disease‐free survival analyses of hub genes were performed using the cBioPortal.*p* < .05 was considered to be of statistical significance

With the help of the cBioPortal database, we analyzed the Hub gene coexpression network (Figure 3A) to initially understand other key genes closely related to these genes, which is useful for analyzing the molecular mechanism of Hub genes in CRC. The biological process network of the hub gene was analyzed with Cytoscape's BiNGO plug‐in (Figure 3B). By hierarchical clustering of hub genes using Xena Functional Genomics Explorer, we found that PHLPP2, ACALB, IGF1, and BCL2 were low‐expressed in colorectal tumor tissues. In contrast, all the other genes were high‐expressed in tumor tissues (Figure 3C). We also analyzed the degree of gene mutation in CRC and clarified the relationship between mutation and survival of CRC patients. By plotting Kaplan–Meier curves, we found that CRC patients with CDCA5, CEP55, HELLS, and NEK2 alterations show worse OS (Figure 4A). Furthermore, CRC patients with CCNB1, cyclin‐dependent kinase 1 (CDK1), CEP55, KIF14, and RFC3 alterations show worse disease‐free survival (Figure 4B).

### 4.3 Transcriptome data analysis CEP55 is upregulated in CRC

**Figure 5:**
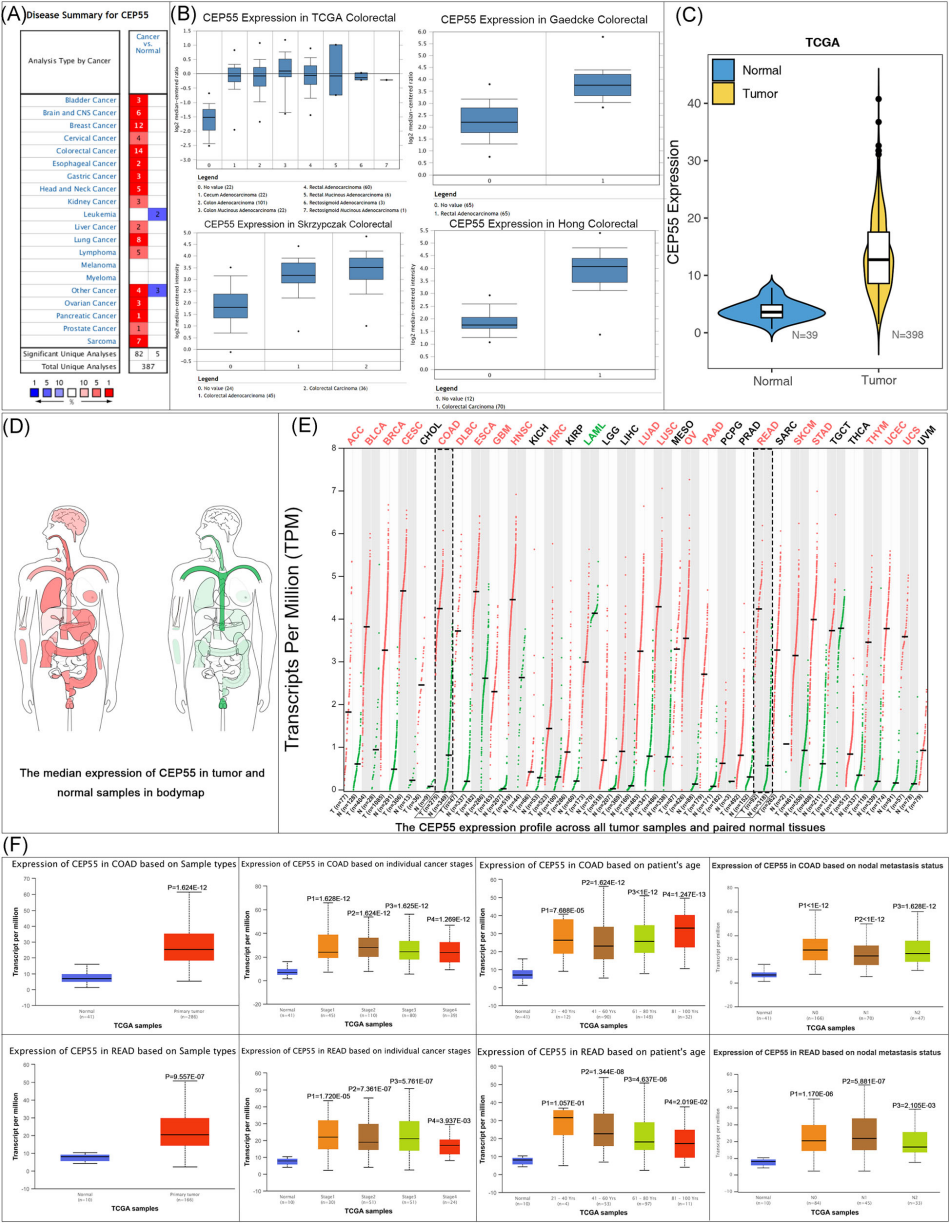
CEP55 is highly expressed in CRC and is associated with multiple clinical factors. (A,B) CEP55 expression in multiple diseases and the expression levels of CEP55 in four CRC datasets (Oncomine). (C) The expression difference of CEP55 in CRC tumor tissue and matching adjacent cancer tissues (sample data from TCGA). (D,E) Expression of CEP55 in extensive sample data and human organs (GEPIA2). (F) The expression of CEP55 was correlated with cancer stage, patient's age, and nodal metastasis status under TCGA samples (UALCAN). CRC, colorectal cancer; TCGA, The Cancer Genome Atlas Program

Analysis in the Oncomine database (https://www.oncomine.org/) found that CEP55 is upregulated in a variety of tumor tissues, such as breast cancer, CRC, and lung cancer. In the four data sets of TCGA colorectal, Gaedcke colorectal,^26^ Skrzypczak colorectal,^27^ and Hong colorectal,^28^ we found that CEP55 expression was higher in tumor tissues than in healthy tissues (Figure 5A,B). We extracted the CEP55 transcriptional information of CRC samples from the TCGA database and found that CEP55 was significantly highly expressed in CRC tumor tissues by *t* test (Figure 5C). Subsequent validation in the GEPIA2 database found the same results, with CEP55 having higher expression in tumor tissues, such as CRC (Figure 5D,E). In further searching for more information about CEP55 in the UALCAN database, we found that the expression of CEP55 was also correlated with patient age, lymph node metastasis, and tumor stage (Figure 5F).

### 4.4 Cell and tissue experiments verified the function of CEP55 in CRC

**Figure 6:**
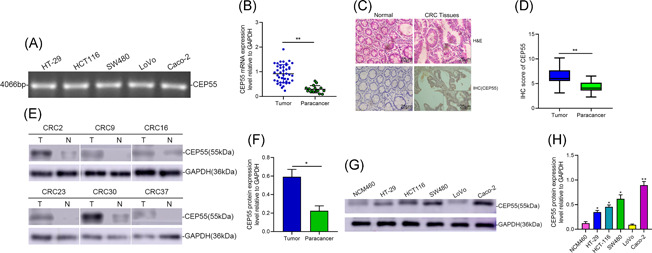
CEP55 is unregulated in human CRC tissues. (A) The DNA expression level of CEP55 has analyzed agarose gel electrophoresis assays. (B) CEP55 mRNA expression was analyzed in CRC samples and the corresponding para cancer tissue samples. (C) Representative images of HE staining and CEP55 staining (brown color) in CRC samples (T) and normal colon tissue (N) (scale bar = 50 μm). (D) IHC scores of tumors and adjacent normal tissues from 37 paired CRC specimens. (E,F) CEP55 protein expression was analyzed by Western blot in 24 human CRC tissues (T) and the corresponding para cancer tissue samples (N) by Western blot analysis. (G,H) Western blot analysis of CEP55 from normal colon cell line (NCM460) and five CRC cell lines. CRC, colorectal cancer; HE, hematoxylin and eosin; IHC, mmunohistochemistry; mRNA, messenger RNA. *p < .05, **p < .01, ***p < .001

**Figure 7:**
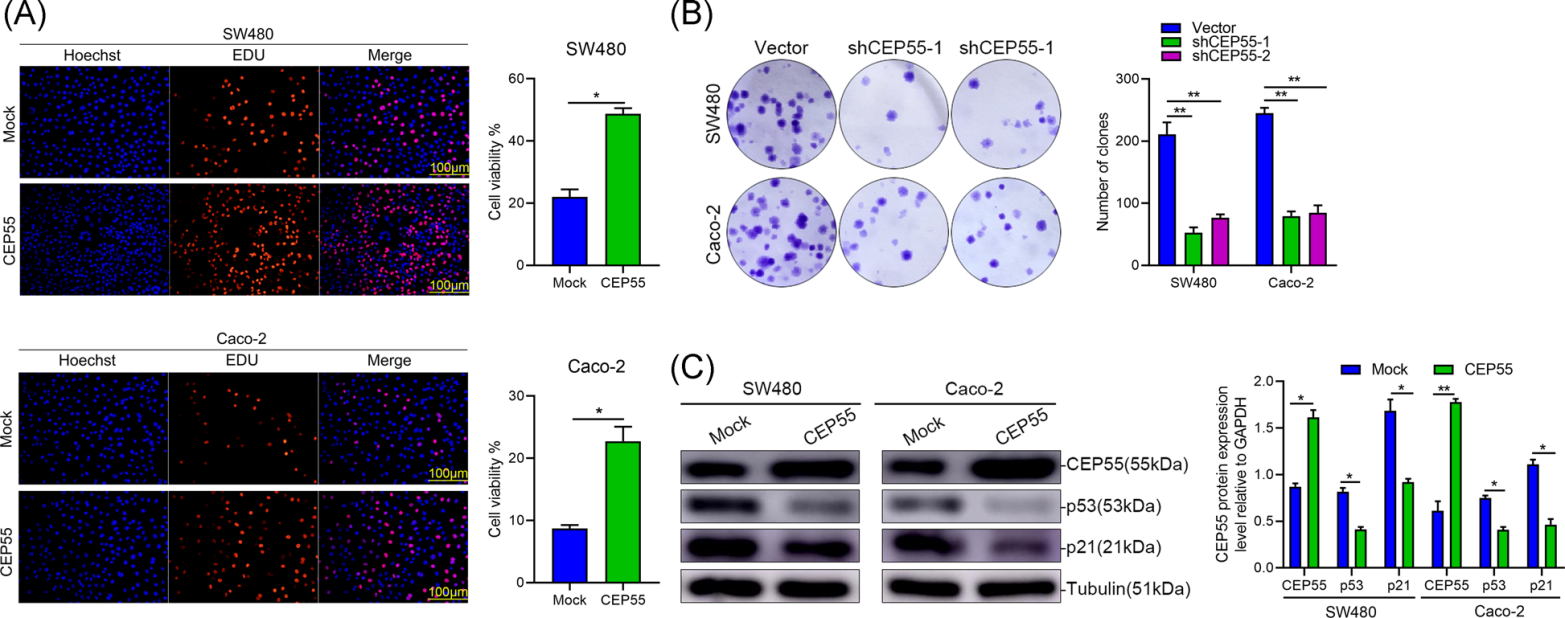
CEP55 promotes the proliferation of CRC cells in vitro by the p53 signaling pathway. (A) Cell proliferation assay was performed by Edu proliferation assays. (B) Colony formation assay after 14 days of culture, with a mean colony counts from three independent experiments. (C) CEP55 regulated the expression of the p53/p21 signaling proteins. Knockdown of CEP55 activated p53/p21 signaling pathway in SW480 and Caco‐2 cells. The protein level of CEP55, p53, and p21 were detected and quantified in CEP55 Knockdowning SW480 and Caco‐2 cells; GAPDH was used as a loading control. CRC, colorectal cancer; GAPDH, glyceraldehyde 3‐phosphate dehydrogenase. *p < .05, **p < .01, ***p < .001

The elevated CEP55 messenger RNA (mRNA) was observed in CRC tissues using RT‐PCR in an OriGene Colon Cancer cDNA array (Figure 6A,B). Immunohistochemical staining was conducted on paired 37 pairs of CRC specimens and healthy colorectal mucosa specimens. The results indicated that CEP55 immunoreactivity was more intense in tumors than in adjacent healthy mucosal tissues (Figure 6C,D) (*p* < .01). As shown in Figure 7E,F, Western blot analysis of CEP55 protein expression in paired 37 pairs of CRC tissues and adjacent tissues from humans indicated that the protein expression level of CEP55 was significantly increased in CRC tissues (*p* < .05). To determine the role of CEP55 in CRC progression, we detected the CEP55 expression in a healthy colon cell line (NCM460) and a series of CRC cell lines (including HT‐29, HCT116, SW480, LoVo, and Caco‐2), and observed higher expression levels of CEP55 in four cell lines (HT‐29, HCT116, SW480, and Caco‐2) (Figure 7G,H) (*p* < .05). These results demonstrate that both mRNA and protein levels of CEP55 were enhanced in human CRC. Proliferation experiments were performed on two cell lines, SW480 and Caco‐2, and the results indicated that the overexpression of CEP55 significantly enhanced the proliferation and metabolism of CRC cells (Figure 7A). Colony formation assay was performed, and the results showed that the growth and colony‐forming ability of CRC cells with silencing CEP55 were significantly lower than the corresponding control cells (*p* < .01) (Figure 7B). To further search for the mechanism involved in the regulation of CRC by high expression of CEP55, we validated the protein expression of p53 and p21 in SW480 and Caco‐2 cells based on our screen p53/p21 signaling pathway, and the results indicated that high CEP55 expression could negatively activate the p53/p21 signaling pathway, thus having an impact on CRC malignant progression (Figure 7C).

## 5 DISCUSSION

In recent years, with the development of personalized medicine and precise medical treatment requirements, a precise diagnosis has become a very urgent project. Various tumor diagnostic biomarkers, such as proteins, exosomes, and hormones have been extensively studied clinically.^29^ According to reports, KRAS, NRAS, and BRAF mutations occur in more than half of CRC patients, and these gene mutations are significantly related to patient survival and can be used to assess patient prognosis.^30^ In this study, we screened and identified CEP55 for the diagnosis of CRC and the prognosis of CRC patients through a combination of bioinformatics and molecular cytology experimental techniques. A regulatory pathway for CEP55 to participate in the regulation of CRC replication and transfer was identified based on the multiple protein pathways identified by the database and the necessary experiments.

With the development of sequencing technology, next‐generation sequencing (NGS) technology has redefined the field of genetic testing. Bioinformatics technology has become an essential part of analyzing sequencing data due to the complexity of sequencing technology.^31^ We used the bioinformatics method to analyze the CRC sequencing data of the GEO database and the TCGA database and preliminarily selected 28 Hub genes. To further understand whether they may play a role in CRC, we analyzed their possible biological processes and pathways involved in regulating tumors. The results show that these genes are mainly involved in regulating the cell cycle and biological processes related to the cell cycle, including DNA repair and other processes. The cell cycle includes processes, such as the replication and separation of genetic material and cell division, usually divided into G0/G1, S, G2, and M phases. The cell cycle is mainly controlled by different CDKs and their functional cyclin partners.^32^ Abnormal activity of various cyclins often leads to uncontrolled proliferation of tumor cells. Based on these shreds of evidence, cell cycle regulators are considered as attractive targets in cancer treatment.^32^, ^33^ Studies have shown that selective autophagy, which participates in maintaining steady‐state processes, regulates cell cycle processes by degrading specific cyclin, regulating cell division, and promoting DNA damage repair to maintain DNA and genomic integrity.^34^ We also found that they are involved in EGFR tyrosine kinase inhibitor resistance and the p53 signaling pathway. EGFR is receptor tyrosine kinases that play essential roles in both normal physiological conditions and cancerous conditions.^35^ After EGFR binds to ligands, both extracellular and intracellular domains of receptor tyrosine kinase undergo dynamic conformational changes, resulting in common phenotypes in tumor cells, such as cell evasion of apoptosis, proliferation, invasion, and metastasis. These altered phenotypes provide the basis for cell carcinogenesis.^36^

Ligand‐dependent activation of EGFR transduces multiple signaling pathways, including the Ras/MAPK pathway, the PI3K/AKT pathway, and the phospholipase C/protein kinase C signaling cascade, which is essential for several cellular functions including survival, proliferation, differentiation, and motility.^37^ The transcription factor p53 is one of the most important tumor suppressor genes currently known, which plays a fundamental role in cell cycle.^38^ In more than 50% of human cancer types, p53 is directly inactivated by mutations, which may lead to tumor progression.^39^ The p53 pathway consists of a network of genes and their products. The goal of these genes and their products is to respond to various endogenous and exogenous stress signals. The p53 protein is activated in a specific way through posttranslational modification, resulting in cell cycle arrest. This is a procedure that induces cell senescence or apoptosis.^40^

In many previous studies, we have learned that mutations accompany the progression of CRC in the APC, K‐Ras, and p53 genes.^41^ The p53 mutation is associated with lymphatic vessel infiltration of the proximal CRC and is significantly associated with the lymphatic and vessel infiltration of the distal CRC.^42^ New evidence from laboratory and clinical trials indicates that some small molecule inhibitors, such as MDM2 protein play an anticancer effect through the reactivation and restoration of p53 function. Compared with wild‐type p53, CRC patients with mutant p53 show higher chemoresistance and have a poorer prognosis.^43^ Taken together, the Hub genes we screened are likely to be able to regulate CRC through related pathways, but sufficient molecular and clinical experiments are needed to explore and verify them before they are clarified.

A single gene does not regulate tumors; many genes work together in tumors through mutual regulation and a series of tumor regulation pathways. In the review by Zhang et al., through bioinformatics analysis, six critical genes involved in CRC transfer, APC, KRAS, BRAF, PIK3CA, SMAD4, and p53, were identified. The mutations of these genes are through various paths and different molecular interactions and regulate the transfer of CRC.^44^ We can see from Figure 2B that there are interactions among 20 proteins, including CCNB1, CEP55, and CDK1, by analyzing the protein interactions between the screened genes. We analyzed the genes that have a coexpression relationship with our Hub genes by predicting co‐expression. In Figure 3A, we can see that many genes have coexpression relationships with these Hub genes. Second, there is the same relationship between Hub genes, such as CDK1 and CEP55, RFC3, and FANCI. These results provide new ideas for future research on the role of related genes in tumors.

In past studies, many genes with significant mutations in CRC have been identified. Rachel Pearlman et al. analyzed the mutations in 25 cancer‐susceptible genes in the DNA of germ cells in 450 patients with CRC diagnosed under the age of 50 using NGS technology. And found 75 gene mutations in 72 patients, every 6 Among the CRC patients diagnosed as under 50 years old, at least one patient has a pathogenic cancer susceptibility gene mutation (16%).^45^ The study of Jun Yu et al. observed seven genes (APC, TP53, KRAS, SMAD4, CDH10, FAT4, and DOCK2) that were repeatedly mutated in CRC. When analyzing the relationship between these gene mutations and the prognosis of CRC patients, one or more of these gene mutations are significantly associated with better OS.^46^ In our study, we analyzed the mutation of 28 Hub genes in CRC patients in cBioportal, grouped patients with these gene mutations and patients without these gene mutations, and analyzed different genes by log‐rank test the relationship between mutation and patient survival time. We found that mutations in CDCA5, CEP55, HELLS, and NEK2 lead to a reduction in OS in patients with CRC (*p* < .05); besides, mutations in CCNB1, CDK1, CEP55, KIF14, and RFC3 are significantly associated with a reduction in disease‐free survival in patients with CRC (*p* < .05). We searched PubMed for the relationship between 8 gene mutations and the prognosis of CRC patients, and there is no research report on the relationship between them.

After searching for relevant literature and understanding the functions of these eight genes, we found that the expression products of these genes are involved in the cell cycle or mitosis and are related to cell proliferation. CEP55 is a coiled‐coil centrosome protein, and a key regulator of cell division, it regulates the organization of mitotic spindles and microtubules and is essential for the cell cycle process.^47^ CEP55 overexpression is related to genomic instability. Murugan Kalimutho et al. have demonstrated through experiments that CEP55 overexpression or knockdown impacts the survival of aneuploid cells. Loss of CEP55 sensitizes breast cancer cells to anti‐mitotic agents through premature CDK1/cyclin B activation and CDK1 caspase‐dependent mitotic cell death. Besides, their study also confirmed that CEP55 is a downstream effector of the MEK1/2‐MYC axis, and the high level of CEP55 mRNA and poor clinical prognosis of breast cancer patients related.^47^ In the study of Chao Jiang et al., immunohistochemical analysis was performed on 203 specimens of primary non‐small–cell lung cancer (NSCLC) and found that CEP55 was upregulated in NSCLC tissues. The overexpression of CEP55 was associated with the poor prognosis of NSCLC patients.^48^ Throughout studies that have continued for many years, it is confirmed that CDK1 and CCNB1 are essential regulators involved in eukaryotic cell mitosis. CDK1 can combine with CCNB1 to form CyclinB1‐Cdk1 kinase. This complex is the active catalytic center of mitosis promoting factor (MPF); its gradual activation can coordinate cells into mitosis.^49^ However, the decrease in MPF activity caused by the dysregulation of CyclinB1–Cdk1 kinase is an essential factor leading to premeiotic block, which is an essential phenotype of cell carcinogenesis.^50^ Recent studies have shown that mitochondria are vital organelles targeted by CDKs. Mitochondrial dysfunction leads to nuclear genome instability, tumorigenesis, tumor growth, therapeutics, and tumor metastasis.^51^ CCNB1/CDK1 can regulate the mitochondrial activity, not only mitochondrial energy output for normal cell cycle progression but also mitochondria‐mediated apoptosis by modifying several prosapoptotic and antiapoptotic proteins when cells are subjected to excessive damage stress.^51^

For several other genes related to the cell cycle, we understand their existing functions. CDCA5 was overexpressed in various tumors, studies have shown that high expression of CDCA5 in CRC may play an essential role in the progression of CRC by activating the ERK signaling pathway and may lead to poor prognosis.^52^ Similar results have been found in patients with lung cancer.^53^ CDCA5 was transcribed by E2F1 in hepatocytes, promotes tumorigenesis by enhancing cell proliferation and inhibiting apoptosis of hepatocellular carcinoma through the AKT pathway, and was significantly related to the poor prognosis of liver cancer patients.^54^, ^55^ Besides, CDCA5 can also be used as a potential therapeutic target for esophageal squamous cell carcinoma.^56^ Overexpression of CDCA5 also predicts a poor prognosis in patients with upper urinary tract and bladder urinary tract cancer.^57^ Recent studies have confirmed that HELLS is significantly upregulated in CRC and is significantly associated with the poor prognosis of CRC patients. HELLS inhibition can lead to cell proliferation, colony production, and G2 + M cell cycle arrest.^58^ In recent years, several studies have shown that NEK2 is overexpressed in CRC, and may affect tumor progression and patient prognosis through various pathways.^59^, ^60^ For KIF14, Wang et al.^61^ have experimentally verified that KIF14 is significantly overexpressed in CRC, and promotes the proliferation of CRC cells and accelerates the cell cycle by activating protein kinase B. KIF14 is also regulated by microRNA‐200c at the posttranscription level. There are many pieces of evidence to prove that RFC3 plays a role in tumors. In lung adenocarcinoma, RFC3 can induce epithelial–mesenchymal transition (EMT) and metastasis of lung adenocarcinoma cells through the Wnt/β‐catenin pathway, and high RFC3 expression may Causes poor prognosis of lung adenocarcinoma.^62^ He et al.^63^ confirmed the overexpression of RFC3 in triple‐negative breast cancer TNBC through cell and animal experiments, and also promoted TNBC metastasis, progression and poor prognosis through EMT signaling pathway. Besides, RFC3 is a candidate carcinogen for esophageal adenocarcinoma. High expression of RFC3 can be used as a poor prognostic indicator, and RFC3 DNA amplification is also prevalent in a variety of epithelial cancer types.^64^ At present, there is no apparent experimental evidence that RFC3 plays a significant role in CRC, but attempts can be made to study the role of RFC3 expression in CRC from the perspective of EMT. These findings also predict to some extent that our findings are relatively reliable and warrant subsequent multi‐directional and multi‐angle studies.

Since our study identified 28 genes that were significantly differentially expressed in CRC, the work required to verify their function was too large, so we selected CEP55 for subsequent analysis. CEP55 mutations in CRC are significantly associated with poor OS and DFS in CRC patients. We found that CEP55 is significantly expressed in CRC (including rectal cancer and colon cancer) in the NGS data of Oncomine and TCGA (*p* < .05). Besides, by analyzing the relationship between CEP55 and some clinicopathological features of colon adenocarcinoma (COAD) and rectum adenocarcinoma (READ) patients, we found that in COAD, the expression of CEP55 in tumor patients over 40 years old increased with age. Nevertheless, in the same age stage of READ patients, the opposite result was found. However, there is no significant evidence that the expression of CEP55 is related to tumor stage and lymph node metastasis.

Because of the possible limitations of pure chip data analysis results, we decided to test the function of CEP55 in CRC through experiments and study‐related mechanisms. According to our experimental results, CEP55 is highly expressed in CRC tissues and cells, and its high expression significantly promotes CRC cell proliferation, migration, and invasion. We also found that high CEP55 expression may negatively activate the p53/p21 signaling pathway, which may Malignant progress has an impact. These findings may have important implications for the diagnosis and treatment of CRC in the future. This is of considerable significance to the prevention and treatment of tumors and to reduce the significant harm of tumors to human society.

Although our research has found some significant results, some shortcomings, such as the number of chip samples we choose may not be enough. Second, the influence of some gene mutations on the prognosis of CRC patients has not been selected for clinical trials and timely follow‐up. Also, we have not conducted in‐depth studies on the specificity and sensitivity of CEP55 as a potential biomarker for CRC. In the future, we should continue to address these issues and continuously improve current research.

In conclusion, we confirmed that CEP55 has high mRNA and protein expression in CRC and mediates the transfer and proliferation of CRC cells, and we have also identified a pathway. CEP55 may be a diagnostic biomarker and a possible prognostic marker in CRC. It can provide valuable suggestions for clinical anticancer treatment, has essential value for the development of new early tumor screening methods, and plays a vital role in predicting tumor prognosis. Besides, we also provide several genes that may play the same role in CRC. These may be potential biomarkers for diagnosis and prognosis in CRC. In the future, we hope to conduct more research on these potential markers.

## CONFLICT OF INTERESTS

The author declare that there are no conflict of interests.

## AUTHOR CONTRIBUTIONS

Kang Lin, Xiaojian Zhu, Chen Luo, and Zhengming Zhu performed the research. Kang Lin and Xiaojian Zhu designed the research study. Fanqin Bu and Jinfeng Zhu contributed essential reagents or tools. Kang Lin, Xiaojian Zhu, and Chen Luo analyzed the data. Kang Lin and Xiaojian Zhu wrote the paper.

## Data Availability

All data are freely available and freely accessible from publicly tumor databases: TCGA (https://cancergenome.nih.gov/), GEO (https://www.ncbi.nlm.nih.gov/geo/), Oncomine (https://www.oncomine.org/resource/login.html).
